# Peroxisome proliferator-activated receptor gamma and spermidine/spermine N^1^-acetyltransferase gene expressions are significantly correlated in human colorectal cancer

**DOI:** 10.1186/1471-2407-6-191

**Published:** 2006-07-19

**Authors:** Michele Linsalata, Romina Giannini, Maria Notarnicola, Aldo Cavallini

**Affiliations:** 1 Laboratory of Biochemistry, National Institute for Digestive Diseases, I.R.C.C.S. "Saverio de Bellis" via Della Resistenza, 70013-Castellana Grotte (BA), Italy

## Abstract

**Background:**

The peroxisome proliferator-activated receptor γ (PPARγ) is a transcription factor that regulates adipogenic differentiation and glucose homeostasis. Spermidine/spermine N^1^-acetyltransferase (SSAT) and ornithine decarboxylase (ODC) are key enzymes involved in the metabolism of polyamines, compounds that play an important role in cell proliferation. While the PPARγ role in tumour growth has not been clearly defined, the involvement of the altered polyamine metabolism in colorectal carcinogenesis has been established. In this direction, we have evaluated the PPARγ expression and its relationship with polyamine metabolism in tissue samples from 40 patients operated because of colorectal carcinoma. Since it is known that the functional role of K-ras mutation in colorectal tumorigenesis is associated with cell growth and differentiation, polyamine metabolism and the PPARγ expression were also investigated in terms of K-ras mutation.

**Methods:**

PPARγ, ODC and SSAT mRNA levels were evaluated by reverse transcriptase and real-time PCR. Polyamines were quantified by high performance liquid chromatography (HPLC). ODC and SSAT activity were measured by a radiometric technique.

**Results:**

PPARγ expression, as well as SSAT and ODC mRNA levels were significantly higher in cancer as compared to normal mucosa. Tumour samples also showed significantly higher polyamine levels and ODC and SSAT activities in comparison to normal samples. A significant and positive correlation between PPARγ and the SSAT gene expression was observed in both normal and neoplastic tissue (r = 0.73, p < 0.0001; r = 0.65, p < 0.0001, respectively). Moreover, gene expression, polyamine levels and enzymatic activities were increased in colorectal carcinoma samples expressing K-ras mutation as compared to non mutated K-ras samples.

**Conclusion:**

In conclusion, our data demonstrated a close relationship between PPARγ and SSAT in human colorectal cancer and this could represent an attempt to decrease polyamine levels and to reduce cell growth and tumour development. Therefore, pharmacological activation of PPARγ and/or induction of SSAT may represent a therapeutic or preventive strategy for treating colorectal cancer.

## Background

The peroxisome proliferator-activated receptor γ (PPARγ) is a member of the nuclear receptor superfamily [1]. It functions as a transcription factor, heterodimerizes with the retinoid X receptor (RXR) and regulates the expression of target genes by binding to the PPAR response elements (PPERs). The PPARγ, initially identified for its role in the metabolism and adipocyte differentiation, is also expressed in tissue outside the adipocyte lineage, including colonic epithelia [2]. In cancer biology PPARγ is the most intensively studied PPAR isoform and several studies have shown the protective role of this nuclear receptor in colorectal tumour [3]. On the contrary, other authors have questioned the antineoplastic activity of PPARγ ligands [4]. In addition, although most of the known target genes activated by PPARγ belong to the pathway of metabolism and lipid transport, the target genes mediating the pro- or anti-cancer activity by affect of the activated PPARγ have not yet been identified.

Recently, Babbar et al. [5] demonstrated that in colorectal tumour cells, the promoter of spermidine/spermine N^1^-acetyltransferase (SSAT) gene contains a functional PPAR responsive element and activated PPARγ is able to activate transcription of this gene.

SSAT and ornithine decarboxylase (ODC) are key enzymes involved in the catabolism and biosynthesis, respectively, of the polyamines (putrescine, spermidine and spermine), compounds that play an important role in both normal and neoplastic cell proliferation [6]. Increased ODC activity and the associated elevation in intracellular polyamines have been implicated in carcinogenesis of many human tissues, including those of the gastrointestinal tract [7-9].

Induction of SSAT plays an important role in lowering the polyamine pool and typically gives rise to growth inhibition as well as exerting tumour-suppressive effects [10,11]. Particularly, Wallace et al. have hypothesized that the increase in SSAT activity may be an attempt to reduce the polyamine pool in tumour cells. [12]

Little is know about the relationship between polyamine metabolism and PPARγ expression in colorectal tumours and available information is derived from in vitro studies [5,13].

We have provided evidence about a close relationship between polyamine biosynthesis and K-ras mutation in human colorectal carcinoma [14]. Moreover, in the human colon adenocarcinoma cell line, it has been shown that mutated K-ras suppressed SSAT via a transcriptional mechanism that involves PPARγ signalling pathway [15].

In order to investigate the relationship between PPARγ and polyamine metabolism in human colorectal carcinoma, the aim of this study was to evaluate the PPARγ, SSAT and ODC gene expression as well as polyamine levels and ODC and SSAT activities, in neoplastic samples and in surrounding uninvolved mucosa. The profile of polyamine metabolism and PPARγ mRNA expression were also investigated in relation to the presence of K-ras mutation in our tumour samples. We have exclusively focalised our attention on mutated codon 12 as it occurs more frequently in human colorectal cancer [16].

## Methods

### Patients

Tissue samples were obtained at the time of surgical resection for colorectal adenocarcinoma from 40 patients: 24 men (mean age 64.8 years, range 36–88) and 16 women (mean age 64.4 years, range 28–87). Pieces of tissue were removed from the peripheral region of the tumour and surrounding mucosa in a region at least 10 cm from the neoplasm. The normal mucosa was found to be macroscopically and microscopically free of neoplastic growth. Immediately after removal, tissue was washed in cold saline solution and then rapidly (within 15–30 min) transported on ice to the laboratory and stored at -80°C until assay. The quality of the tumour and surrounding normal tissue specimens and the relative cellular composition were determined by histopathological assessment. The clinical and histopathological features of colorectal cancer patients are reported in Table 1. The design of this study was approved by the local ethics committee.

Informed consent was obtained from all patients before their surgery and examination of specimens used in this study.

### Analysis of the PPARγ, ODC and SSAT gene expression

PPARγ, ODC and SSAT mRNA levels were evaluated by reverse transcriptase and real-time PCR. The RNA isolation, reverse transcriptase and real-time PCR (RT-PCR) methods have been described previously [17]. In brief, after RNA extraction, aliquots of 5 μl of the reverse transcriptase reaction (20 μl), corresponding to 0.5 μg of total RNA, were subjected to the RT-PCR in 25 μl of final volume contained master mix (iQ SYBR Green Supermix, BioRad, Milan, Italy). The amplification program on a iCycler iQ Real-Time detection system (BioRad) was: 30 s at 95°C and then 10 s at 94°C, 10 s at 55°C and 30 s at 72°C for 45 cycles. A further melt-curve step (melt-curve started at 55°C and the temperature was increased 0.5°C per cycle for 80 cycles) was added in the thermocycler program at the end of the last extension phase of 45 cycles to control the presence of the secondary non specific PCR products or primer-dimer products. For quantitative analysis the external standard curves were used and the mRNA levels of each target gene were then normalized to those of the β-actin, as control gene.

The primers used were: 5'-AGGCGAGGGCGATCTTGACA-3' (forward primer) and 5'-ACCAGGAATGCTTTTGGCATACTCT-3' (reverse primer) for PPARγ (GenBak: NM_138712); 5'-GGTCCGCAAAGGGAAGAAA-3' and 5'-TGCCAATCCACGGGTCATA-3' for SSAT (GenBank: NM_002970); 5'-GATTCCAAAGCAGTCTGTCGTCTCA-3' and 5'-TTGTCCAACGCTGGGTTGATTACGC-3' for ODC (GenBank: M16650); 5'-AAGACCTGTACGCCAACACAGTGCTGTCTGG-3' and 5'-CGTCATACTCCTGCTTGCTGATCCACATCTGC-3' for β-actin (GenBank: NM_001101). The relative mRNA levels are expressed as target gene/β-actin ratio.

### Polyamine analysis

Each tissue sample (approximately 10–15 mg) was homogenized in 700 μl of 0.9 percent sodium chloride mixed with 5 μl (174 μM) of the internal standard 1, 10-Diaminodecane (1, 10-DAD). In order to precipitate the proteins, 50 μl of perchloric acid (PCA) 3 M were added to the homogenate. After 30 minutes incubation in ice, the homogenate was centrifuged for 15 minutes at 7000 g. The supernatant was filtered (Millex-HV_13 _pore size 0.45 μm, Millipore, Bedford, MA) and lyophilized. The residue was dissolved in 250 μl of HCl (0.1 M). Aliquots (100 μl) were reacted with dansyl chloride and the dansyl-polyamine derivatives were determined by high performance liquid chromatography (HPLC) as previously described [7]. Polyamine levels were expressed as concentration values in nmol/g wet weight tissue.

### ODC activity

ODC activity was measured with a radiometric technique as previously described [14]. In brief, the reaction mixture consists of DL-[1-^14^C]-ornithine and tissue extract in Tris-HCL buffer; the ^14^CO_2 _product generated by the enzyme reaction was trapped on filter paper pre-treated with NaOH, which was suspended in a center well above the reaction mixture. Radioactivity on the filter papers was determined by a liquid scintillation counter (model 1219 Rackbeta, LKB-Pharmacia, Uppsala, Sweden). ODC activity was expressed as pmol CO_2_/h/mg of protein.

### SSAT activity

SSAT activity was measured according to the method described by Wallace et al. [18] with minor modifications. The reaction was started by adding 70 μL of cytosol to a microfuge tube containing 30 μL of incubation buffer (30 mM spermidine, 0.1 μCi [acetyl-1-^14^C] acetyl coenzyme A, 1 M Tris-HCL, pH 7.8). The incubation was carried out for 10 min at 37°C, then 20 μL hydroxylamine hydrochloride were added to stop the reaction. The tubes were boiled for 3 min with pierced tops and then spun in a microfuge for 5 min. A quantity of 30 μL of supernatant was spotted on to Whatman P81 discs which were then washed five times with distilled water and three times with 100% ethanol. The radioactivity on the filter papers was determined by a liquid scintillation counter. SSAT activity was expressed as pmol of N^1^-[^14^C] acetylspermidine generated/min/mg of protein.

### K-ras mutation analysis

Genomic DNA was isolated from 50 mg of normal mucosa and tumour tissue by TRI-Reagent (Mol. Res. Centre Inc. Cincinnati, OH, USA) following manufacturer's instruction. The extracted DNA was dissolved in 50 μl of sterile water and used for PCR. Mutations in K-ras exon 1, codon 12 were screened by PCR amplification followed by RFLP analysis as previously described [14].

### Statistical analysis

Differences among polyamine levels, ODC activity, SSAT activity, and mRNA expression of relative genes were analyzed by Wilcoxon matched pairs test and Mann Whitney test when appropriate. The significance of differences, for the above parameters, in location of tumour, Dukes'stages, and differentiation grade was determined by Kruskal-Wallis analysis of variance. Correlations were evaluated by Spearman correlation test. All data are expressed as mean ± SEM. A p-value < 0.05 was considered as significant.

## Results

**Figure 1:**
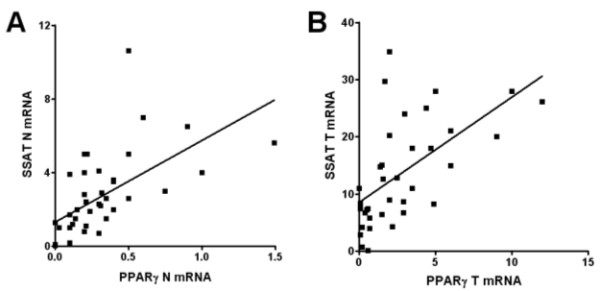
Representative correlations between PPARγ and SSAT mRNA expression in colorectal tissue. **A**. PPARγ mRNA expression correlates with that of SSAT mRNA levels in the normal mucosa samples of 40 patients (r = 0.73, p < 0.0001; Spearman correlation coefficient). **B**. PPARγ mRNA expression correlates with that of SSAT mRNA levels in the tumour tissue samples of 40 patients (r = 0.65, p < 0.0001, Spearman correlation coefficient). The relative mRNA levels are expressed as target gene/β-actin ratio.

The PPARγ, ODC and SSAT mRNA levels were significantly higher in colorectal carcinoma than in normal surrounding mucosa. In addition, polyamine levels, ODC and SSAT activities were significantly increased in neoplastic samples than in normal ones (p < 0.05; Wilcoxon matched pairs test) (Table 2). Of note, a significant and positive correlation between PPARγ and SSAT gene expression was observed in both normal and neoplastic tissue (r = 0.73, p < 0.0001; r = 0.65, p < 0.0001, respectively; Spearman correlation coefficient) (Figure 1).

K-ras codon 12 mutation was found in 12 out of 40 patients (30%). PPARγ expression, as well as ODC and SSAT mRNA levels were significantly higher (p < 0.05; Mann Whitney test) in colorectal carcinoma samples expressing K-ras mutation as compared to non mutated K-ras samples. Mutated K-ras tissues also showed significantly higher (p < 0.05) polyamine levels, as well as ODC and SSAT activities, as compared to non mutated K-ras tissues (Table 3).

The gene expression, the polyamine levels and the enzymatic activities were not associated with age, sex, tumour site, histological differentiation, and Dukes' stage (data not shown).

## Discussion

Several studies have described increased or decreased levels of PPARγ expression in different human tumour tissue as compared to normal mucosa [19-21]. Hence, taking into consideration the diversity of human cancer, the expression of PPARγ may likely be dependent on tissue specificity and/or mutational events that are prerequisite for cancer development. In our study, real time RT-PCR analysis demonstrates a significant enhancement in the expression of PPARγ in colorectal tumours as compared to normal surrounding mucosa. Few studies have investigated the expression of PPARγ in human colorectal carcinoma in relation to normal mucosa. Previously, the comparison of PPARγ expression levels in 11 human colon adenocarcinomas revealed no change in comparison to normal mucosa [22]. In a recent study an increase of PPARγ expression in comparison to normal mucosa it has been observed in 25% (four) of patients with colorectal tumour [23] These contrasting results could be attributed to differences in methodological procedures as well as to the number of patients analyzed. With regard to the role of PPARγ in colon cancer, the available data are conflicting and mostly obtained in cell lines or in animal models [24-27]. On the other hand, it is well known that polyamine metabolism is an integral component of the mechanism of colorectal carcinogenesis [9,28]. In this study, a close and positive correlation was observed between the PPARγ and SSAT expression both in normal and neoplastic human colorectal tissues. Studies performed with colorectal tumour cell lines have shown that cells transfected with the PPARγ restored the SSAT promoter activity, and an activated PPARγ could increase SSAT expression in these cells [15]. Thus, our findings in human tissue are compatible with the *in vitro *studies about the transcriptional induction of PPARγ on the SSAT gene.

In the light of these data, we suggest that, in colorectal normal mucosa, PPARγ induces SSAT expression and influences polyamine availability, regulating cell proliferation and differentiation. In fact, it is known that the requirement of polyamines in cell growth is typically met by a biosynthetic pathway regulated by ODC and balanced by a polyamine catabolic pathway regulated by SSAT [29].

In colorectal tumour tissue, we observed an increase in PPARγ and SSAT expression as well as in SSAT activity.

The relationship between SSAT and cancer is only now being defined. In this respect, in vitro and in vivo studies are consistent with the notion that SSAT suppresses cell growth and tumour development [30,31]. On the contrary, a Min mouse model in which SSAT expression promotes tumorigenesis has been proposed. [32]. Furthermore, the role of SSAT in the polyamine metabolism in the cancer tissue seems to be tissue dependent. In fact, it has been observed that the increase in SSAT activity leads to polyamine pool depletion and growth inhibition in breast tumour [12,33].

On the other hand, in prostatic cancer the SSAT overexpression induces growth inhibition without determining polyamine pool depletion since SSAT activity seems to modulate ODC activity [11,34].

In this study, we report both higher polyamine levels and ODC activity in neoplastic tissue samples than in normal ones, thus confirming our previous data and those of other groups obtained in human colorectal cancer or in other types of carcinoma [35-39].

We have also found that while colon cancer tissue exhibits about a 13-fold increase of the ODC activity in comparison to normal tissue, SSAT activity rises by 1.5-fold only. The tumour polyamine pool seems to be maintained at higher levels by a robust increase in ODC activity not properly balanced by a severe increase in SSAT activity. Therefore, the SSAT response could be secondary to a pro-cancerous increase in ODC activity and polyamine levels. In this direction, we can suppose that the increase in polyamine catabolic activity, as a result of PPARγ induction without a concomitant downregulation of the polyamine biosynthetic pathway, could be insufficient for counteracting tumour development.

Our findings about PPARγ and polyamine metabolism in mutated K-ras colorectal cancer seem to follow this direction. In fact, we have detected higher levels of PPARγ and SSAT expression, as well as higher SSAT activity, in mutated K-ras samples. These data suggest that in cancer tissue with high rate of proliferation and likely with enhanced polyamine levels and ODC activity [14], there is an attempt to restore a growth control via SSAT with a mechanism involving PPARγ. However, in a vitro study, it has been shown that mutated K-ras suppressed the SSAT expression via a transcriptional mechanism involving the PPARγ signalling pathway [15]. The in vitro findings may not be directly relevant to the in vivo effects and different tumour models can result in diversified functional outcome.

## Conclusion

In conclusion, our data show a close relationship between PPARγ and SSAT in human colorectal cancer and this could represent an attempt to decrease polyamine levels and to reduce cell growth and tumour development. These findings support the hypothesis that pharmacological activation of PPARγ and/or induction of SSAT may represent, in future, an effective therapeutic or preventive strategy for colorectal cancer. In this direction a therapeutic or chemopreventive approach using a combination of polyamine biosynthesis inhibitor like difluoromethylornithine (DFMO), and an agent, like a non-steroidal anti-inflammatory drug (NSAID) which induces catabolism, should be more efficacious than a single approach. This rationale has provided the basis of two ongoing chemoprevention studies [40].

## Competing interests

The author(s) declare that they have no competing interests.

## Authors' contributions

ML conceived the study, participated in the manuscript preparation and performed polyamine analysis and enzymatic activities assay. MN carried out the K-ras mutation analysis. RG carried out the tissue collection and real-time PCR analysis. AC, participated in the study design and in the manuscript preparation and elaborated the gene expression analysis. All authors have read and approved the final manuscript.

## Pre-publication history

The pre-publication history for this paper can be accessed here:



## Figures and Tables

**Table 1:** Clinical and histopathological features of colorectal cancer patients (40 cases)

	No of patients
*sex*	
male	24
female	16
Location of tumour*	
right	14
left	14
rectum	12
*Dukes' stage*	
A	2
B	13
C	15
D	10
*Differentiation grade§*	
G1	10
G2	18
G3	12

*Right colon: caecum, ascending colon, hepatic flexure, transverse colon; *left colon*: splenic flexure, descending colon and sigmoid. § *G1*: well-differentiated, *G2*: moderately differentiated, *G3*: poorly differentiated.

**Table 2:** Polyamine levels, enzymatic activities and genes expression in normal and neoplastic colorectal tissues

	Normal	Neoplastic	p
Putrescine	18.74 ± 3.1	50.87 ± 10.5	0.001
Spermidine	150.1 ± 10.6	302.2 ± 27.8	<0.0001
Spermine	387.3 ± 27.98	538.0 ± 41.7	0.003
Total polyamines	556.2 ± 39.9	891.0 ± 70.7	0.0002
ODC	21.2 ± 3.5	274.2 ± 70.9	<0.0001
SSAT	27.4 ± 3.8	40.4 ± 5.0	0.030
ODC mRNA	12.5 ± 2.0	20.7 ± 5.0	0.020
SSAT mRNA	2.7 ± 0.3	13.6 ± 1.5	<0.0001
PPARγ mRNA	0.3 ± 0.05	2.8 ± 0.5	<0.0001

Polyamines are reported as nmol/g wet tissue. ODC activity is expressed as pmol ^14^CO_2_/h/mg of protein. SSAT activity is expressed as pmol N^1^-[^14^C] acetylspermidine/min/mg of protein. The relative mRNA levels are expressed as target gene/β-actin ratio. All data are presented as mean ± SEM (Wilcoxon matched pairs test).

**Table 3:** Polyamine levels, enzymatic activities and genes expression in neoplastic colorectal tissue with and without K-ras mutation.

	Mutated K-ras n = 12	Non mutated K-ras n = 28	p
Putrescine	64.4 ± 14.7	41.0 ± 14.5	0.039
Spermidine	376.1 ± 49.2	248.8 ± 26.9	0.021
Spermine	652.2 ± 48.6	455.5 ± 55.99	0.020
Total polyamines	1093.0 ± 98.0	745.3 ± 85.5	0.012
ODC	525.1 ± 157.6	92.9 ± 26.5	0.002
SSAT	43.1 ± 5.6	25.92 ± 4.3	0.035
ODC mRNA	21.3 ± 4.4	8.79 ± 1.8	0.020
SSAT mRNA	18.0 ± 3.9	7.3 ± 1.4	0.011
PPARγ mRNA	2.9 ± 1.1	0.78 ± 0.2	0.032

Polyamines are reported as nmol/g wet tissue. ODC activity is expressed as pmol ^14^CO_2_/h/mg of protein. SSAT activity is expressed as pmol N^1^-[^14^C] acetylspermidine/min/mg of protein. The relative mRNA levels are expressed as target gene/β-actin ratio. All data are presented as mean ± SEM (Mann Whitney test).

## References

[B1] Rosen ED, Spiegelman BM (2001). PPARγ: a nuclear regulator of metabolism, differentiation, and cell growth. J Biol Chem.

[B2] Lefebvre A-M, Paulweber B, Fajas L, Woods J, McCrary C, Colombel J-F, Najib J, Fruchart J-C, Datz C, Vidal H, Desreumaux P, Auwerx J (1999). Peroxisome proliferator-activated receptor gamma is induced during differentiation of colon epithelium cells. J Endocrinol.

[B3] Koeffler HP (2003). Peroxisome proliferator-activated receptor γ and cancers. Clin Cancer Res.

[B4] Auwerx J (2002). Nuclear receptors. PPARγ in the gastrointestinal tract: gain or pain?. Am J Physiol Gastrointest Liver Physiol.

[B5] Babbar N, Ignatenko NA, Casero RA, Gerner EW (2003). Cyclooxygenase-independent induction of apoptosis by sulindac sulfone is mediated by polyamines in colon cancer. J Biol Chem.

[B6] Thomas T, Thomas TJ (2003). Polyamine metabolism and cancer. J Cell Mol Med.

[B7] Linsalata M, Caruso MG, Leo S, Guerra V, D'Attoma B, Di Leo A (2002). Prognostic value of tissue polyamine levels in human colorectal carcinoma. Anticancer Res.

[B8] Notarnicola M, Linsalata M, Caruso M, Caruso ML, Altomare D, Lorusso D, Leo S, Di Leo A (2003). Genetic and biochemical changes in colorectal carcinoma in relation to morphologic characteristics. Oncol Rep.

[B9] Milovic V, Turchanowa L (2003). Polyamines and colon cancer. Biochem Soc Trans.

[B10] Seiler N (2004). Catabolism of polyamines. Amino Acids.

[B11] Kee K, Foster BA, Merali S, Kramer DL, Hensen ML, Diegelman P, Kisiel N, Vujcic S, Mazurchuk RV, Porter CW (2004). Activated polyamine catabolism depletes acetyl-CoA pools and suppresses prostate tumor growth in TRAMP mice. J Biol Chem.

[B12] Wallace HM, Duthie J, Evans DM, Lamond S, Nicoll KM, Heyes SD (2000). Alterations in polyamine catabolic enzymes in human breast cancer tissue. Clin Cancer Res.

[B13] Takashima T, Fujiwara Y, Higuchi K, Arakawa T, Yano Y, Hasuma T (2001). PPAR-γ ligands inhibit growth of human esophageal adenocarcinoma cells through induction of apoptosis, cell cycle arrest and reduction of ornithine decarboxylase activity. Int J Oncol.

[B14] Linsalata M, Notarnicola M, Caruso MG, Di Leo A, Guerra V, Russo F (2004). Polyamine biosynthesis in relation to K-ras and p-53 mutations in colorectal carcinoma. Scand J Gastroenterol.

[B15] Ignatenko NA, Babbar N, Mehta D, Casero RA, Gerner EW (2004). Suppression of polyamine catabolism by activated Ki-ras in human colon cancer cells. Mol Carcinog.

[B16] Bleeker WA, Hayes VM, Karrenbeld A, Hofstra RM, Verlind E, Hermans J, Poppema S, Buys CH, Plukker JT (2001). Prognostic significance of K-ras and TP53 mutations in the role of adjuvant chemotherapy on survival in patients with Dukes C colon cancer. Dis Colon Rectum.

[B17] Giannini R, Cavallini A (2005). Expression analysis of a subset of coregulators and three nuclear receptors in human colorectal carcinoma. Anticancer Res.

[B18] Wallace HM, Evans DM, David ML Morgan (1998). Measurement of spermidine/spermine N^1^-acetyltransferase activity. Methods in molecular biology.

[B19] Karger S, Berger K, Eszlinger M, Tannapfel A, Dralle H, Paschke R, Fuhrer D (2005). Evaluation of peroxisome proliferator-activated receptor-gamma expression in benign and malignant thyroid pathologies. Thyroid.

[B20] Zhang GY, Ahmed N, Riley C, Oliva K, Barker G, Quinn MA, Rice GE (2005). Enhanced expression of peroxisome proliferator-activated receptor gamma in epithelial ovarian carcinoma. Br J Cancer.

[B21] Therashita Y, Sasaki H, Haruki N, Nishiwaki T, Ishiguro H, Shibata Y, Kudo J, Konishi S, Kato J, Koyama H, Kimura M, Sato A, Shinoda N, Kuwabara Y, Fujii Y (2002). Decreased peroxisome proliferator-activated receptor gamma gene expression is correlated with poor prognosis in patients with esophageal cancer. Jpn J Clin Oncol.

[B22] Sarraf P, Mueller E, Jones D, King FJ, DeAngelo DJ, Partridge JB, Holden SA, Chen LB, Singer S, Fletcher C, Spiegelman BM (1998). Differentiation and reversal of malignant changes in colon cancer through PPARγ. Nat Med.

[B23] Feilchenfeldt J, Brundler MA, Soravia C, Totsch M, Meier CA (2004). Peroxisome proliferator-activated receptors (PPARs) and associated transcription factors in colon cancer: reduced expression of PPARγ-coactivator 1 (PGC-1). Cancer Lett.

[B24] DuBois R, Gupta R, Brockman J, Reddy BS, Krakow SL, Lazar MA (1998). The nuclear eicosanoid receptor, PPARγ, is aberrantly expressed in colonic cancers. Carcinogenesis.

[B25] Lefebvre AM, Chen I, Desreumaux P, Naijb J, Fruchart JC, Geboes K, Briggs M, Heyman R, Auwerx J (1998). Activation of the peroxisome proliferator activated receptor gamma promotes the development of colon tumors in C57BL/6J-APCMin/+mice. Nat Med.

[B26] Shimava T, Kojima K, Yoshiura K, Hiraishi H, Terano A (2002). Characteristics of peroxisome proliferators-activated receptor gamma (PPARγ) ligand induced apoptosis in colon cancer cells. Gut.

[B27] Kohno H, Suzuki R, Sugie S, Tanaka T (2005). Suppression of colitis-related mouse colon carcinogenesis by a COX-2 inhibitor and PPAR ligands. BMC Cancer.

[B28] Linsalata M, Russo F, Notarnicola M, Guerra V, Cavallini A, Clemente C, Messa C (2005). Effects of genistein on the polyamine metabolism and cell growth in DLD-1 human colon cancer cells. Nutr and Cancer.

[B29] Thomas T, Thomas TJ (2001). Polyamines in cell growth and cell death: molecular mechanisms and therapeutic applications. Cell Mol Life Sci.

[B30] Chen Y, Kramer DL, Jell j, Vujcic S, Porter CW (2003). Small interfering RNA suppression of polyamine analog-induced spermidine/spermine N1-acetyltransferase. Mol Pharmacol.

[B31] Kee K, Vujcic S, Merali S, Diegelman P, Kisiel N, Powell CT, Kramer DL, Porter CW (2004). Metabolic and antiproliferative consequences of activated polyamine catabolism in LNCaP prostate carcinoma cells. J Biol Chem.

[B32] Tucker JM, Murphy JT, Kisiel N, Diegelman P, Barbour KW, Davis C, Medda M, Alhonen L, Janne J, Kramer DL, Porter CW, Berger FG (2005). Potent modulation of intestinal tumorigenesis in Apcmin/+ mice by the polyamine catabolic enzyme spermidine/spermine N1-acetyltransferase. Cancer Res.

[B33] Vujcic S, Halmekyto M, Diegelman p, Gan G, Kramer DL, Janne J, Porter CW (2000). Effects of conditional of overexpression of spermidine-spermine N1-acetyltransferase on polyamine pool dynamics, cell growth, and sensitivity to polyamine analogs. J Biol Chem.

[B34] Kee K, Vujcic S, Merali S, Diegelman P, Kisiel N, Powell CT, Kramer DL, Porter CW (2004). Metabolic and antiproliferative consequences of activated polyamine catabolism in LNCaP prostate carcinoma cells. J Biol Chem.

[B35] Linsalata M, Russo F, Cavallini A, Berloco P, Di Leo A (1993). Polyamines, diamine oxidase, and ornithine decarboxylase activity in colorectal cancer and in normal surrounding mucosa. Dis Colon Rectum.

[B36] Linsalata M, Cavallini A, Di Leo A (1997). Polyamine oxidase activity and polyamine levels in human colorectal cancer and in normal surrounding mucosa. Anticancer Res.

[B37] Weiss T, Bernhardt G, Buschauer A, Thasler WA, Dolgner D, Zirngibl H, Jauch KW (2002). Polyamine levels of human colorectal adenocarcinomas are correlated with tumor stage and grade. Int J Colorectal Dis.

[B38] Naso P, Lantieri R, Acquaviva R, Licata F, Bonanno G, Licata A (2005). Polyamines levels in colorectal cancer: new markers?. Hepatogastroenterol.

[B39] Russo F, Linsalata M, Giorgio I, Caruso ML, Armentano R, Di Leo A (1997). Polyamine levels and ODC activity in intestinal-type and diffuse-type gastric carcinoma. Dig Dis Sci.

[B40] Basuroy UK, Gerner EW (2006). Emerging concepts in targeting thepolyamine metabolic pathway in epithelial cancer chemoprevention and chemotherapy. J Biochem.

